# The Associations between Depressive Symptoms and Self-Rated Health in Relation to Sense of Coherence among Adolescents: Cross-Sectional Study

**DOI:** 10.3390/children11101244

**Published:** 2024-10-16

**Authors:** Vilija Malinauskiene, Romualdas Malinauskas

**Affiliations:** Department of Physical and Social Education, Lithuanian Sports University, 44221 Kaunas, Lithuania

**Keywords:** adolescents, depressive symptoms, self-rated health, sense of coherence

## Abstract

Background: We investigated the predictors of poor SRH in a representative sample of Lithuanian mainstream school students in grades 7–8. We also checked for gender differences in the associations between SRH and depressive symptoms and other predictors. Methods: A total of 2104 7th–8th-grade students participated (response rate 73.95%) and were asked about depressive symptoms, psychosomatic health complaints, negative acts at school, feeling at school, family stress and violence, sense of coherence, self-esteem, and lifestyle. We used a hierarchical regression analysis including a variety of self-rated health predictors. Results: Boys scored significantly higher on physical activity and smoking, whereas girls scored significantly higher on SRH, depressive symptoms, psychosomatic health complaints, and family stress and violence, though the significance was lost in the hierarchical regression. Depressive symptoms were the strongest predictor of poor SRH (standardized *β* = 0.309, *p* < 0.001), though other investigated predictors were also significant but had lower effect sizes. Strong evidence was found supporting the buffering role of sense of coherence in the relationship between depressive symptoms and SRH (standardized *β* = −0.266, *p* < 0.001). Conclusions: We can conclude that the magnitude of the relationship between depressive symptoms and self-rated health is dependent on the levels of sense of coherence. We did not find gender differences in those associations. As poor SRH is easy to determine, especially with a one-item question, the cases of poorly rated health should be detected early and corrected by interventions in order to prevent poor health outcomes in the future.

## 1. Introduction

Self-rated health (SRH) is a subjective measure in which individuals assess their own health status. Adolescents conceptualize health as a construct related to medical, psychological, social, and lifestyle factors [1]. It is commonly used in health research as a reliable predictor of overall health and well-being. Despite the seemingly non-specific nature of SRH, it has been shown to be an unusually strong predictor of mortality [2]. When it comes to adolescents, SRH is a valuable tool for understanding their perception of their own health and can provide insights into their physical, mental, and social well-being [3]. Emotional and psychological well-being plays a crucial role in SRH. Adolescents may consider factors like stress, anxiety, depression, and overall mental wellness when assessing their health [4].

The use of an SRH questionnaire captures a holistic view of health among adolescents. Adolescent appraisals of their health are shaped by their overall sense of functioning, which includes physical health and non-physical health dimensions, as well as psychological distress [5]. There is a clear association between SRH in adolescence and the likelihood of developing multiple or chronic diseases in early adulthood [6]. Notably, individuals who report poor SRH in adolescence face a markedly increased risk of developing such diseases. It is important to pay close attention to SRH during adolescence, as it is a predictor of future health problems and a means to implement targeted preventive measures. Therefore, it is essential to expand the research in this area to better understand the specific predictors and factors influencing SRH.

The relationship between adolescent SRH and depressive symptoms is a significant area of research in the fields of psychology, psychiatry, and public health. Depressive symptoms, such as persistent sadness, loss of interest, changes in sleep patterns, and feelings of worthlessness, can significantly impact an adolescent’s mental health, though only a few studies investigated the associations between depressive symptoms and SRH. A study among university students indicated that students experiencing depressive symptoms may be more likely to perceive their overall health negatively [7]. Another study among adolescents investigated the impact of intrapersonal, interpersonal, and environmental factors on the trajectories of self-rated health and depressive symptoms [8].

Psychosomatic complaints, which are physical symptoms with no clear medical explanation, can significantly impact adolescents’ SRH. These complaints may include headaches, stomachaches, muscle tension, and fatigue, among others. Research suggests that psychosomatic complaints are common during adolescence, possibly due to the myriads of physical, psychological, and social changes occurring during this period. Adolescents who experience frequent psychosomatic complaints often report lower SRH compared to their peers [9]. Persistent physical symptoms can lead to a decreased sense of well-being and overall health perception [10].

The relationship between adolescent SRH and family stress and violence is a critical area of study that involves understanding how the family environment, particularly stressors and violence, can impact adolescents’ perceptions of their own health. Adolescents who experience family stress and violence may be more likely to report lower SRH due to the psychological trauma associated with such experiences. Traumatic events within the family can affect an adolescent’s overall well-being and contribute to a negative self-perception and low SRH [11]. Family stress and violence can disrupt the normal functioning of family dynamics and support systems. Higher levels of emotional, physical, and sexual abuse, as well as physical neglect, were significantly associated with poorer ratings of health [12]. Adolescents may feel a lack of emotional support and a breakdown in their social connections, negatively influencing their SRH. On the other hand, good parent–adolescent relationships are associated with significantly higher levels of self-rated general health in young adulthood [13]. The relationship between adolescent SRH and engagement in negative acts at school is an important area of study that involves understanding how behaviors such as bullying, aggression, or other negative acts can impact adolescents’ perceptions of their own health. Adolescents who are victims of negative acts at school may experience adverse mental health outcomes, including increased stress, anxiety, and depression. Such experiences can contribute to a negative self-perception of health [14].

The relationship between adolescents’ SRH and self-esteem is an important area of study that explores how individuals’ perceptions of their overall health relate to their sense of self-worth and confidence. Research suggests a positive association between higher SRH and elevated self-esteem among adolescents. When individuals perceive themselves as being in good health, it may contribute to a more positive overall self-concept [15].

Regular physical activity (PA) is frequently linked to improved self-rated health among adolescents [16]. PA has numerous physical and mental health benefits, including increased self-esteem, enhanced mood, and reduced stress. An international study involving 36 countries showed empirical evidence for the connection between SRH and intense PA in teenagers [17]. The study [17] shows a positive correlation between SRH and intense PA. Zhang et al. [18] analysis revealed a connection between PA and increased SRH, as well as between sedentary behavior (SB) and lower SRH in PA in teenagers. Notably, a ‘dose–response’ link has been established for SHR and PA, with higher PA levels correlating with better SRH compared to lower PA levels. This relationship was consistent across genders, with no significant gender disparities observed in the relationship of PA, SB, and SRH between boys and girls.

Research on adolescents’ self-rated health particularly focuses on their willingness to start using alcohol. The relationship between adolescents’ self-rated health and alcohol use is a complex and important area of study, as it involves understanding how adolescents’ perceptions of their own health are linked to their alcohol consumption. Alcohol use can have a direct impact on adolescents’ SRH. Drinking alcohol excessively may trigger various health problems, such as liver disease, impaired cognitive function, and increased vulnerability to accidents and injuries, all of which can influence how adolescents perceive their health. Some adolescents may turn to alcohol as a coping mechanism for dealing with stress or emotional challenges. The use of alcohol to cope with stressors can negatively impact both physical and mental health, influencing SRH [19]. Concerning smoking, research evidence suggests that active or passive smoking may have an impact on teenagers’ SRH [20].

Antonovsky’s model suggests that individuals with a strong sense of coherence (SOC) perceive life events as comprehensible, controllable, and relevant. A low sense of coherence reflects general anxiety as well as ongoing depressive symptoms [21], and a strong sense of coherence leads to increased well-being. Teenagers with a higher SOC may experience less anxiety and depression, which can contribute to a more positive SRH.

Adolescence marks a period characterized by a wide range of changes, physical, emotional, and social, and understanding the interplay between self-perception of health and depressive symptoms is essential for supporting young people’s mental health [1]. Adolescents with a higher SOC may be better equipped to make sense of their health status, manage stressors, and find meaning in their experiences, which can influence their SRH positively [22]. Therefore, SOC might show a potential moderating role in the association between depression and SRH.

We hypothesize that depression symptoms in adolescents might play a crucial role in them negatively assessing their health and that other possible predictors of SRH (psychosomatic health complaints, family stress and violence, negative behaviors in school, lifestyle factors like alcohol consumption and physical activity, and internal features like self-esteem and SOC) can even increase the associations between depression and SRH in adolescents. We also presume that SOC might act as a moderator in the associations between depression and SRH.

One objective of this study was to investigate gender differences in SRH predictors. Another objective was to investigate the correlations between SRH and all the predictors that were included in the study. As we hypothesized that the strongest predictor of SRH among adolescents might be depressive symptoms, the third objective was to investigate the associations between depressive symptoms and SRH and, further, to study the relative importance of each predictor in relation to others. Thus, we aimed to assess the dynamics of the strongest predictor concomitantly with four blocks of variables: external (negative acts at school, family stress and violence, and feeling at school), internal (self-esteem and sense of coherence), lifestyle (physical activity, smoking, and alcohol), and, as other researchers suggest that adolescents with a higher SOC may experience lower levels of depression, which can contribute to a more positive SRH [22], the final block was supplemented with interaction between depressive symptoms and SOC.

## 2. Materials and Methods

### 2.1. Study Design

The present study was designed on the basis of a cross-sectional sample of 2104 adolescents, investigating the predictors of SRH from secondary schools in Lithuania in 2023 by a self-administered questionnaire on internal (depressive symptoms, SOC, self-esteem, and psychosomatic complaints about health), external (family stress and violence and negative acts at school), and lifestyle (alcohol consumption, smoking, and physical activity) factors.

### 2.2. Study Participants and Procedure

According to the Official Statistics portal of Lithuania, in 2018, there were 230 lower secondary education schools with 19000 7th–8th grade students in Lithuania. A total of 30 secondary schools were chosen at random from a register provided by the Education, Science, and Sport Ministry. This research received approval from the University’s Ethics Committee. All students and their parents were provided with detailed instructions on the aim of the survey, and the students participated in the research with their parents’ informed consent. In a total of ninety-seven 7th–8th grade classes, 2845 students have been surveyed, and 2104 fully completed questionnaires (response rate 73.95%) have been included in the analyses. Some students did not respond because they did not want to take part in the survey at the time of data collection or because they did not go to school. In the sample, 50.4% were girls, and 49.6% were boys.

### 2.3. Indicators of Self-Rated Health and Feeling at School

Self-rated health (SRH) was evaluated using a single-item five-choice question (“How do you rate your health in general”: “Excellent”, “Good”, “Average”, “Fair”, “Poor”). Previous studies have found that single-question health measurement is suitable for use in other teenage health investigations [23]. In this study, boys rated their health better than girls (*p* < 0.01) (Table 1). Feeling at school was assessed by the question “How do you feel at school”, using five response options from ”I am very bad at school” to “I am very good at school” [23].

### 2.4. Depressive Symptoms

We used the pediatric depressive symptoms (patient-reported outcomes measurement information system or PROMIS) scale, consisting of 8 items [24]. The person was asked to rate his/her feelings over the last seven days. The items included “I felt sad”, “I felt lonely”, “It was hard for me to have fun”, etc. The response choices were from “never” to “almost always”. A total score was computed by summing up all the items, with higher scores reflecting more depressive symptoms. This scale was selected based on its relative brevity, its appropriateness for use with a wide age range of youth, and its promising evidence of reliability and validity [25]. In this study, internal consistency reliability was measured with a Cronbach α coefficient of 0.92.

### 2.5. Self-Esteem

Self-esteem was evaluated using the Rosenberg scale [26]. This scale includes 10 questions with four possible answers, from ”strongly agree” to “strongly disagree”, which were then rated 1, 2, 3, or 4 points, respectively, with five items scored in the opposite direction. The total score was counted (ranging from 10 to 40), with higher scores representing poorer self-esteem. The scale demonstrated good intra-consistency, as assessed by Cronbach’s α, with a value of 0.771.

### 2.6. Sense of Coherence

Antonovsky stressed the holistic nature of the SOC scale [27]. This tool covers the key components of a sense of coherence: meaningfulness, comprehensibility, and manageability. Participants selected an option from a 5-point Likert-type scale for each of the items, ranging from 1 to 5. In this study, the overall SOC-13 score is a summation of the answers to each survey question and varies in the range of 13 to 65, with higher scores representing a greater degree of SOC. The SOC-13 Cronbach’s α in this study was 0.894. 

### 2.7. Psychosomatic Health Complaints

Psychosomatic health complaints have been measured using HBSC survey questions [28]. Participants had to answer how frequently they experienced the following psychosomatic symptoms in the previous six months: “feeling dizzy”, “feeling tense”, “sleeplessness”, “get up tired in the morning”, “stomach ache and back pain”, and “headache”. Every symptom could be rated with one of the following options: 1 = “rarely or never”, 2 = “about every month”, 3 = “about every week”, 4 = “more than once a week”, or 5 = “about every day”. The responses have been aggregated, and scores for psychosomatic complaints have been provided. More severe symptoms of psychosomatic health complaints were reflected in higher scores. The internal consistency of the measure was 0.863.

### 2.8. Family Stress and Violence

One single question has been applied to measure family stress, “Do you feel calm and satisfied at home?”, with 4 possible answers: “I feel calm at home”, “I rarely experience conflicts in the family”, “often conflicts in the family”, “permanent tension in the family” [28]. Family violence has been measured using a one-item question, “Do you sustain violence in your family (beatings, mocking, humiliation, or threat)?” with the following options for responses: “never”, “experienced 1–2 times in my life”, “experience 2–3 times a month”, “experience once a week”, “experience several times per week”. The responses to the questions on domestic violence and stress were aggregated to form a continuous variable, where higher scores reflect greater levels of the indicator.

### 2.9. Negative Acts at School

Negative behavior in school was measured [29] by asking a series of items about different forms of negative behavior that students may have experienced in the last few months (isolation or social exclusion, verbal bullying, threatening or forcing someone to do something, sexual bullying, physical bullying, bullying through lies and false rumors, stealing or damaging money or other items, etc.) with one of the following options for responses: “it hasn’t happened to me in the past couple of months”, “only once or twice”, “two or three times a month”, “about once a week”, “several times a week”. The overall score was computed after aggregating all the responses and creating a continuous measure of negative school behavior.

### 2.10. Lifestyle

One question proposed by the WHO was used to measure leisure-time physical activity [30]: “How often in leisure time have you been physically active (sports, running, etc.), no less than 60 min in a way that your breathing becomes hard and sweat appears” with seven possible answers: “every day”, “4–6 times per week”, “2–3 times per week”, “once a week”, “2–3 times per month”, “a few times per year and less often”, and “unable to exercise due to illness”, where higher values represent poorer PA.

One statement was used to determine the level of smoking: “At this moment, how often do you smoke?”. The answer choices and their scores were as follows: “I have never smoked” = 4, “I smoked some time before, but I quit” = 3, “I occasionally smoke” = 2, and “I smoke at least one cigarette a day” = 1, with higher scores reflecting higher rates of tobacco use. The students were asked to indicate their alcohol consumption with one of the following options for responses: “I do not drink at all”, “I drink 2–3 times a year”, “I drink occasionally”, “I drink each month”, and “I drink once a week or more frequently”, where a higher score reflects a more severe level of alcohol consumption.

### 2.11. Statistical Analysis

Statistical analysis was performed with SPSS 28.0. First, the variables were tested for a normal distribution, and then Pearson correlations between variables were calculated. Girls and boys were compared using the independent samples t-test. A hierarchical linear regression analysis has been carried out with SRH as the dependent variable and five blocks of predictors, as we wanted to add or remove variables from our models in multiple steps. Block 1 consisted of depressive symptoms and psychosomatic health complaints and gender (gender is not presented in the table), block 2 added negative experiences (family stress and violence and negative acts at school) and self-esteem, block 3 expanded with internal variables (self-esteem and sense of coherence), block 4 with lifestyle variables, and finally, the fifth block was supplemented with interaction between depressive symptoms and SOC. Gender was included in all blocks and showed no statistical significance. Statistical significance was determined with a *p*-value threshold of less than 0.05.

## 3. Results

Table 1 shows the distribution of study variables across gender groups. Analyzing the data by gender, there are 1060 (50.4%) girls and 1044 (49.6%) boys. The mean age of the whole sample was 14.62 ± 1.61 years, 14.68 ± 1.60 years for boys and 14.55 ± 1.61 years for girls.

Mean gender differences for the variables are presented in Table 1. The boys had significantly higher scores for PA and smoking, while the girls had higher scores for SRH, depressive symptoms, psychosomatic health complaints, and family stress and violence.

The computed Pearson correlations among the research indicators are presented in Table 2.

SRH showed a significant positive correlation with all survey parameters, with the strongest relationship being with depressive symptoms (0.498, *p* < 0.01): the higher the levels of depressive symptoms, the more negatively health was rated. We identified positive correlations of SRH with negative acts at school (0.329, *p* < 0.01), psychosomatic health complaints (0.324, *p* < 0.01), self-esteem (0.322, *p* < 0.01), family stress and violence (0.304, *p* < 0.01), and lower PA (0.206, *p* < 0.01). The analyses revealed a negative correlation between feeling at school (−0.230, *p* < 0.01) and SOC (−0.307, *p* < 0.01): the higher the SOC, the more positively health was rated.

In Table 3, we report the strength of the association between the covariates and the dependent variable (SRH) in the hierarchical regression analysis, where the standardized value of beta is interpreted as the effect size, and R-squared is the explained variance. Depressive symptoms was the strongest predictor of SRH, with a standardized beta of 0.309 and an R-squared of 0.295 of the whole model IV, which included psychosomatic health complaints, self-esteem, negative acts at school, family stress and violence, PA, alcohol, and sense of coherence. Thus, more depressive symptoms, more psychosomatic health complaints, more negative acts at school and family stress and violence, lower self-esteem, lower levels of PA, higher levels of alcohol use, and a lower SOC were predictive of higher rates of negative SRH symptoms among teenagers.

SOC was a strong predictor of SRH alone, but in the final model V, a significant interaction effect was found between depressive symptoms and sense of coherence on SRH, with a standardized beta of −0.266, showing that the strength of the relationship between depressive symptoms and SRH is linked to the SOC indicator. Other predictors listed in Table 3 were significant as well, but their effect sizes were definitely low. Gender and smoking showed no significant effect in any of the models.

## 4. Discussion

As SRH is an indicator of health status that integrates a wide range of factors, in this study, we have analyzed different indicators, including depressive symptoms, psychosomatic health complaints, negative acts at school, feeling at school, family stress and violence, sense of coherence, self-esteem, and lifestyle [31]. Poor SRH ‘consistently predicts reduced longevity, even when objective disease conditions and risk factors are considered’ [32] (p. 1). Despite the fact that many studies have already been conducted on SRH among adolescents, there are still unanswered questions, especially on the interaction effects of several predictors, e.g., depressive symptoms and SOC. In our study, we used a hierarchical regression analysis including a variety of SRH predictors among the adolescents in a representative sample of the Lithuanian 7th–8th-grade students of secondary schools.

Previous research has demonstrated that gender differences exist in SRH among adolescents. Girls, in some cases, may be more likely to report lower SRH compared to boys. Gender is an important factor, and girls with health problems are more likely to experience poorer SRH [33]. Good mental health leads to a greater variety of participation in social factors [34]. However, in our study, no gender differences in SRH were confirmed. The strongest predictor of SRH in our study was depressive symptoms; others were psychosomatic health complaints, negative acts at school, feeling at school, self-esteem, family stress and violence, PA, and alcohol. Our findings coincide with other recent investigations [7,11,14,15,33].

SOC is a concept introduced by sociologist Aaron Antonovsky, representing a person’s general orientation toward life, coping abilities, and the perceived comprehensibility, manageability, and meaningfulness of stressful situations. Adolescents with a strong SOC are thought to be more resilient in the face of stressors. A higher SOC may contribute to effective coping strategies, aiding adolescents in dealing with health challenges and promoting a positive SRH [35]. It is reasonable to argue that SOC is an important source of health enhancement that builds resilience and positive subjective health status. A study from Norwegian adolescents aged 13–19 years ‘provided support for the significant role of SOC as a coping resource, especially in relation to adolescents’ mental health; weaker associations were found with subjective health complaints and SRH’ [22] (p. 8).

Our study indicates that the strength of the relationship between depressive symptoms and SRH depends on the level of SOC, therefore supporting the implementation of SOC strengthening measures among adolescents at school, especially taking into account that, according to Antonovsky, SOC develops until the age of thirty years, remains relatively constant until pension age, and declines in old age [27].

Adding an interaction term to a linear model—estimated using regression—becomes necessary when the statistical association between a predictor and an outcome depends on the value/level of another predictor. We included the interaction of depressive symptoms and sense of coherence in the fifth hierarchical regression model. When considering the interaction effects of depressive symptoms and sense of coherence, SOC has been found to play a defensive role in preventing SRH. We can conclude that the magnitude of the association between depression symptoms and SRH is dependent on the level of SOC.

Our previous study among adolescents in the city of Kaunas found significant predictors of depressive symptoms; the strongest among them was SOC. The current study was arranged with the purpose of investigating the predictors of SRH in a large representative sample of Lithuanian adolescents, as poor SRH is easier to detect, especially with one-item questions. This approach could be used for prevention purposes, as youth is a time of energy, streaming mental capacities, and vitality, and, therefore, the cases of poorly rated health should be detected early and corrected by interventions in order to prevent poor health outcomes in the future.

The strengths of this study include having a representative sample of Lithuanian secondary school students and the fact that the results can be generalized as reliable for the general population of students. Another strong point was definitely the high response rate and the application of an adequate statistical method (hierarchical regression) that allowed us to reveal the predictors of SRH at different levels. Limitations include the use of self-administered questionnaires, the probable bias, and the use of a cross-sectional study type that limits us to make a causal inference. Longitudinal studies would be more reliable, with the possibility of causal relationship determination in a representative sample. Cross-sectional studies that rely on people to respond to questionnaires are particularly prone to non-response bias. We avoided such bias by excluding non-responses from the study.

## 5. Conclusions

The present study was prepared to reveal the predictors of SRH among students of secondary schools in Lithuania as well as gender differences in the associations. Boys scored significantly higher on physical activity and smoking, whereas girls scored significantly higher on SRH, depressive symptoms, psychosomatic health complaints, and family stress and violence, though the statistical significance of the difference between genders was lost in the hierarchical regression. The findings from the hierarchical regression analysis confirmed that depressive symptoms were the strongest predictor of negative SRH, though other investigated predictors, such as psychosomatic health complaints, negative acts at school, feeling at school, self-esteem, SOC, family stress and violence, and lifestyle, were also significant, but with lower effect sizes. Another finding in our study was that when considering the interaction effects of depressive symptoms and SOC, significant support for a protective/buffering role of SOC was found in relation to SRH. We did not find gender differences in those associations.

## Figures and Tables

**Table 1:** Statistics for study questionnaires divided between girls and boys.

	Total (N = 2104)	Girls (N = 1060)	Boys (N = 1044)	*t*	*p*	Cohen’s *d*
Self-rated health	1.82 ± 0.89	1.85 ± 0.84	1.78 ± 0.94	1.69	<0.001 **	0.27
Feeling at school	3.47 ± 0.87	3.59 ± 0.87	3.36 ± 0.85	6.19	0.09	0.27
Depressive symptoms	16.67 ± 5.82	17.22 ± 5.99	16.10 ± 5.60	4.41	<0.001 **	0.19
Psychosomatic health complaints	14.22 ± 6.99	14.87 ± 6.85	13.56 ± 7.07	4.33	<0.001 **	0.19
Negative acts at school	12.66 ± 4.69	12.49 ± 4.24	12.83 ± 5.11	−1.67	0.09	−0.07
Family stress and violence	2.74 ± 1.05	2.83 ± 0.97	2.66 ± 1.12	3.64	<0.001 **	0.16
Sense of coherence	44.07 ± 9.86	44.15 ± 9.22	43.99 ± 10.47	0.35	0.73	0.02
Self-esteem	19.63 ± 4.73	19.47 ± 4.87	19.79 ± 4.57	−1.55	0.12	−0.07
Smoking	1.37 ± 0.83	1.33 ± 0.78	1.41 ± 0.87	−2.32	0.02 *	−0.10
Alcohol	2.01 ± 1.17	1.96 ± 1.09	2.06 ± 1.26	−1.79	0.07	−0.08
Physical activity	3.04 ± 1.71	3.55 ± 1.71	2.52 ± 1.56	14.32	<0.001 **	0.63

* *p* < 0.05; ** *p* < 0.001.

**Table 2:** Correlations of study variables.

	1	2	3	4	5	6	7	8	9	10	11	12
1. Self-rated health	1											
2. Gender	−0.069 **	1										
3. Feeling at school	−0.230 **	−0.130 **	1									
4. Depressive symptoms	0.498 **	−0.094 **	−0.330 **	1								
5. Psychosomatic health complaints	0.324 **	−0.086 **	−0.168 **	0.576 **	1							
6. Negative acts	0.329 **	0.036	−0.252 **	0.527 **	0.290 **	1						
7. Family stress and violence	0.304 **	−0.069 **	−0.128 **	0.398 **	0.238 **	0.330 **	1					
8. Self-esteem	0.322 **	0.030	−0.179 **	0.467 **	0.258 **	0.247 **	0.197 **	1				
9. Smoking	0.097 **	0.051 *	−0.085 **	0.078 **	0.177 **	0.038	0.165 **	−0.143 **	1			
10. Alcohol	0.112 **	0.039	−0.071 **	0.082 **	0.153 **	0.047 *	0.202 **	−0.144 **	0.449 **	1		
11. Physical activity	0.206 **	−0.298 **	−0.082 **	0.220 **	0.077 **	0.062 **	0.127 **	−0.100 **	0.106 **	0.060 **	1	
12. Sense of coherence	−0.307 **	−0.008	0.243 **	−0.611 **	−0.434 **	−0.355 **	−0.277 **	−0.459 **	−0.143 **	−0.139 **	−0.101 **	1

* p < 0. 05; ** *p* < 0.01.

**Table 3:** Predictors of negative SRH among adolescents (hierarchical regression analysis).

Variable			*β*		
	Model I	Model II	Model III	Model IV	Model V
Depressive symptoms	0.467 **	0.361 **	0.331 **	0.309 **	0.636 **
Psychosomatic health complaints	0.055 *	0.057 *	0.066 **	0.064 **	0.057 *
Negative acts at school		0.066 *	0.069 **	0.080 *	0.060 *
Family stress and violence		0.116 **	0.116 **	0.098 **	0.094 **
Feeling at school		−0.0 69 **	−0.0 68 **	−0.0 63 **	−0.0 66 **
Sense of coherence			−0.56 *	−0.0 59 *	−0.2 76 **
Self-esteem			0.124 **	0.128 **	0.131 **
Smoking				0.015	0.014
Alcohol				0.046 *	0.046 *
Physical activity				0.099 **	0.100 **
Depressive symptoms × Sense of coherence					−0.2 66 **
R-square	0.250	0.271	0.283	0.295	0.303
R-square change		0.021 **	0.011 **	0.013 **	0.008 **

* *p* < 0.05, ** *p* < 0.001.

## Data Availability

The datasets collected and analyzed during the current study are available from the corresponding author upon reasonable request.
